# Reduced adiponectin levels in patients with COVID‐19 acute respiratory failure: A case‐control study

**DOI:** 10.14814/phy2.14843

**Published:** 2021-04-27

**Authors:** Sean M. Kearns, Katelyn W. Ahern, James T. Patrie, William B. Horton, Thurl E. Harris, Alexandra Kadl

**Affiliations:** ^1^ Department of Medicine University of Virginia School of Medicine Charlottesville VA USA; ^2^ Department of Pharmacology University of Virginia School of Medicine Charlottesville VA USA; ^3^ Eli Lilly and Company Indianapolis IN USA; ^4^ Division of Biostatistics Department of Public Health Sciences University of Virginia School of Medicine Charlottesville VA USA; ^5^ Division of Endocrinology and Metabolism University of Virginia School of Medicine Charlottesville VA USA; ^6^ Division of Pulmonary and Critical Care Medicine University of Virginia School of Medicine Charlottesville VA USA

**Keywords:** adipokines, adiponectin, COVID‐19, critical illness, metabolism, respiratory insufficiency

## Abstract

Hypoadiponectinemia is speculated to play a key role in the relationship between obesity and COVID‐19 respiratory failure. However, only one study has examined adiponectin levels in COVID‐19 patients, and none have investigated adiponectin levels strictly in patients with acute respiratory failure. In this study, we performed a retrospective case‐control study of adipokine levels in patients with acute respiratory failure caused by either COVID‐19 or other viral/bacterial source. All patients with COVID‐19 respiratory failure in the University of Virginia Biorepository and Tissue Research database were included. We also selected patients with non‐COVID‐19 infectious respiratory failure from the same biorepository to serve as a comparison cohort. Plasma adipokine levels were measured on three occasions during the first 72 hours of hospitalization. Twelve patients with COVID‐19 respiratory failure and 17 patients with other infectious respiratory failure were studied. Adiponectin levels were significantly lower in patients with COVID‐19 respiratory failure, even after adjustment for age, sex, BMI, and other covariates. In conclusion, adiponectin levels appear to be reduced in COVID‐19 respiratory failure. Larger studies are needed to confirm this report.

**Abbreviations**
APACHE: Acute Physiology and Chronic Health Evaluation
BMI: body mass index
COVID‐19: coronavirus disease 2019
PAI‐1: plasminogen activator‐inhibitor‐1
SOFA: Sequential Organ Failure Assessment
UVA: University of Virginia

## 1 INTRODUCTION

Obesity is a major risk factor for hospitalization, respiratory failure, and mortality in patients with coronavirus disease 2019 (COVID‐19) (Frank et al., 2020; Anderson et al., 2020), but the mechanisms underlying this are poorly understood. The anti‐inflammatory adipokine adiponectin has been speculated to play a role in COVID‐19 respiratory failure (Lockhart & O'Rahilly, 2020), but only one study has examined adiponectin levels in patients with COVID‐19 (Zelst et al., 2020). No studies have examined adiponectin levels strictly in patients with COVID‐19 acute respiratory failure.

## 2 MATERIALS AND METHODS

We included all patients with COVID‐19 respiratory failure who consented to donate blood samples to the University of Virginia (UVA) Intensive Care Unit (ICU) Biorepository. We then selected patients with non‐COVID‐19 respiratory failure (i.e., due to other viral or bacterial infections) in the UVA ICU Biorepository to serve as a comparison cohort. We followed the Strengthening the Reporting of Observational Studies in Epidemiology (STROBE) guidelines and our study complied with all principles outlined in the Declaration of Helsinki. All study protocols were approved by the UVA Institutional Review Board for Health Sciences Research (Protocol #21101). Respiratory failure was defined as patients with acute respiratory distress syndrome using Berlin criteria (Force et al., 2012) who were on mechanical ventilation in the ICU. COVID‐19 diagnoses were confirmed by RealTime SARS‐CoV‐2 assay (Abbott Molecular Inc.; Des Plaines, IL). Plasma samples for adipokines were obtained on ICU admission and then 24 and 72 hr later. Human adipokine (i.e., adiponectin, adipsin, resistin, lipocalin‐2, and plasminogen activator‐inhibitor‐1 (PAI‐1)) levels in plasma were measured using the Milliplex Human Adipokine Magnetic Bead Panel 1 kit (Millipore Sigma; Burlington, Massachusetts) following manufacturer's protocol. Plasma samples were prepared at a 1:400 dilution. If analytes were found to be above the linear range, samples were run again with a 1:40,000 dilution. Hour 0, 24, and 72 adipokine measurements were analyzed on the natural logarithmic scale as repeated measures by way of linear mixed models. Hypothesis testing, with respect to comparing the measurement distributions of the aforementioned list of outcome variables between the COVID‐19 and other infectious respiratory failure cohorts, was conducted in a hierarchical manner. For each outcome variable, a global null hypothesis test was first conducted to test the null hypothesis that independent of the hour of blood draw, the geometric mean of the COVID‐19 respiratory failure cohort outcome variable measurement distribution did not differ from the geometric mean of the other infectious respiratory failure outcome variable measurement distributions. If the global null hypothesis was rejected at the 0.05 significance level, then a second set of hypothesis tests were conducted to identify at what hour of blood draw the geometric mean of the COVID‐19 respiratory failure outcome variable measurement distribution differed from the geometric mean of the other infectious respiratory failure outcome variable distribution at the 0.05 significance level. Results were adjusted for the following covariates: age, sex, body mass index (BMI), preexisting diabetes mellitus, enteral nutrition, glucocorticoid therapy, total insulin, Acute Physiology and Chronic Health Evaluation (APACHE) II score, and Sequential Organ Failure Assessment (SOFA) score. Bonferroni correction was applied to adjust for multiple comparisons. All statistical analyses were performed using Statistical Analysis System (SAS) version 9.4 (SAS Institute; Cary, NC).

## 3 RESULTS

In total, twenty‐nine patients were included in this study. Table 1 provides descriptive statistics for subjects in both the COVID‐19 (n = 12) and the non‐COVID‐19 (n = 17) infectious respiratory failure cohorts. Notably, the two cohorts were generally similar in baseline characteristics and clinical outcomes. For the non‐COVID‐19 acute respiratory failure group, infectious diagnoses included presumed bacterial pneumonia (n = 8), presumed hospital‐acquired pneumonia (n = 1), presumed healthcare‐associated pneumonia (n = 1), pseudomonas aeruginosa (n = 1), influenza A (n = 4), parainfluenza (n = 1), and human metapneumovirus (n = 1).

**TABLE 1:** Baseline demographic characteristics and clinical outcome data for study cohorts. Data are presented as mean ± standard deviation where appropriate. For the non‐COVID‐19 acute respiratory failure group, infectious diagnoses included presumed bacterial pneumonia (n = 8); presumed hospital‐acquired pneumonia (n = 1); presumed healthcare‐associated pneumonia (n = 1); Pseudomonas aeruginosa (n = 1), influenza A (n = 4), parainfluenza (n = 1), and human metapneumovirus (n = 1). Non‐intubated patients were managed with either high‐flow nasal cannula oxygen supplementation or noninvasive positive pressure ventilation

Variable	COVID−19 Respiratory Failure (n=12)	Non‐COVID−19 Respiratory Failure (n=17)
Age, years	61.3 ± 16.0	61.4 ± 16.9
Sex, n (% Male)	9 Male; 3 Female (75%)	9 Male; 8 Female (53%)
Mean BMI (kg/m^2^)	32.8 ± 9.5	30.2 ± 5.4
Pre‐Existing Diabetes Mellitus, n (%)	6 (50%)	8 (47%)
Mean Admission APACHE II Score	13.6 ± 5.0	14.6 ± 7.0
Mean Admission SOFA Score	5.0 ± 3.3	4.2 ± 3.1
P/F Ratio	128 ± 62	173 ± 87
Intubated, n (%)	10 (83%)	14 (82%)
Length of Intubation, days	12.6 ± 7.0	10.7 ± 10.7
Length of Stay, days	21.4 ± 13.4	20.0 ± 21.1
Alive at Discharge, n (%)	9 (75%)	14 (82%)

Abbreviations: APACHE, Acute Physiology and Chronic Health Evaluation; BMI, body mass index; P/F Ratio, arterial pO2/fraction of inspired oxygen (i.e., FiO2); SOFA, Sequential Organ Failure Assessment.

**FIGURE 1:**
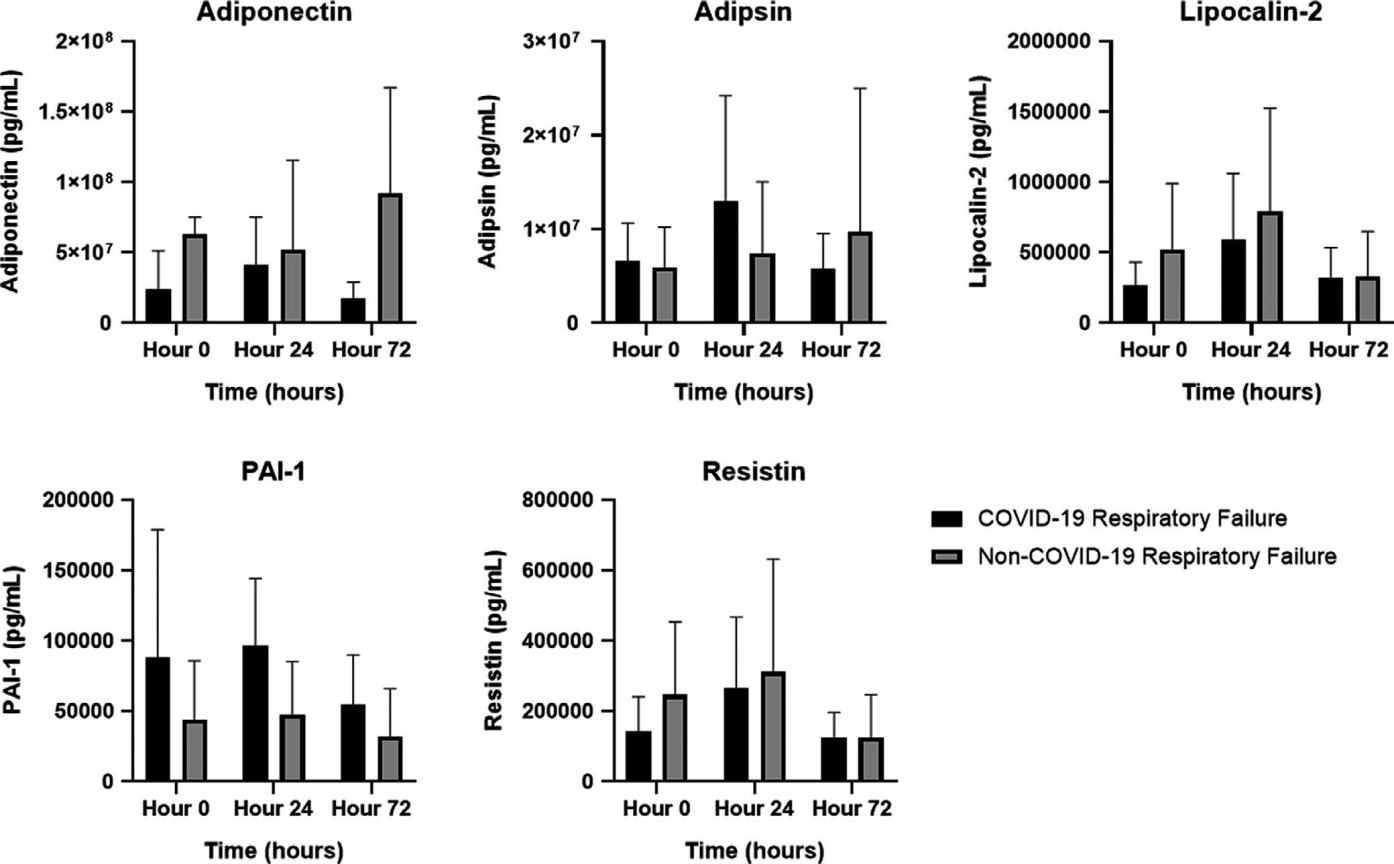
Summary statistics (mean and standard deviation) for adipokine levels within the COVID‐19 and non‐COVID‐19 (i.e., other infectious) respiratory failure cohorts

Table 2 and Figure 1 detail statistical analyses of and summary statistics for adipokine levels within each cohort during the first 72 hr of ICU admission. Adipsin, resistin, lipocalin‐2, and PAI‐1 did not significantly differ between groups at any single time point or globally over the 72 hr of evaluation. Conversely, adiponectin levels were significantly lower in the COVID‐19 cohort over the 72 hr of evaluation (*p *= 0.003), as well as being significantly lower at Hour 0 (*p *= 0.031) and Hour 72 (*p *= 0.004). Adiponectin levels remained significantly lower in those with COVID‐19 after adjustment for age, sex, BMI, pre‐existing diabetes mellitus, enteral nutrition, glucocorticoid therapy, total insulin, APACHE II score, and SOFA score (Figure 1).

**TABLE 2:** Daily and global geometric mean ratios for measured adipokines. Geometric mean ratio represents Non‐COVID‐19 (i.e., other infectious) Respiratory Failure:COVID‐19 Respiratory Failure. All comparisons were adjusted for age, sex, BMI, enteral nutrition, preexisting diabetes mellitus, glucocorticoid therapy, APACHE II score, SOFA score, and total insulin. Unadjusted estimates are provided first, followed by adjusted estimates in parentheses

Adipokine	Hour	Geometric Mean Ratio	Lower 95% CI	Upper 95% CI	*p*‐value	Bonferroni Lower 95% CI	Bonferroni Upper 95% CI	Bonferroni *p*‐value	Global *p*‐value
Adiponectin^a^	0	2.87 (2.58)	1.23 (1.10)	6.68 (6.09)	0.015 (0.031)	1.01 (0.89)	8.15 (7.47)	0.046 (0.093)	0.001 (0.003)
	24	0.90 (0.75)	0.41 (0.32)	1.96 (1.76)	0.787 (0.503)	0.34 (0.26)	2.35 (2.14)	1.000 (1.000)	
	72	4.41 (3.73)	2.10 (1.62)	9.25 (8.61)	<0.001 (0.004)	1.75 (1.31)	11.10 (10.62)	0.001 (0.011)	
Adipsin^a^	0	1.08 (1.81)	0.61 (0.94)	1.91 (3.51)	0.799 (0.075)	0.53 (0.80)	2.18 (4.12)	1.000 (0.226)	0.325 (0.330)
	24	0.84 (1.44)	0.44 (0.75)	1.60 (2.76)	0.586 (0.270)	0.38 (0.64)	1.87 (3.21)	1.000 (0.809)	
	72	1.15 (1.82)	0.54 (0.94)	2.45 (3.49)	0.695 (0.071)	0.45 (0.80)	2.98 (4.14)	1.000 (0.212)	
Lipocalin−2^b^	0	2.21 (2.04)	1.07 (0.93)	4.54 (4.47)	0.032 (0.074)	0.91 (0.77)	5.39 (5.40)	0.096 (0.222)	0.158 (0.144)
	24	1.27 (1.14)	0.66 (0.53)	2.45 (2.48)	0.463 (0.726)	0.57 (0.44)	2.85 (2.98)	1.000 (1.000)	
	72	0.88 (0.80)	0.48 (0.37)	1.63 (1.70)	0.677 (0.539)	0.41 (0.31)	1.89 (2.07)	1.000 (1.000)	
PAI−1^c^	0	0.74 (1.01)	0.40 (0.44)	1.38 (2.29)	0.337 (0.987)	0.35 (0.36)	1.59 (2.78)	1.000 (1.000)	0.019 (0.234)
	24	0.47 (0.69)	0.26 (0.31)	0.83 (1.52)	0.011 (0.349)	0.23 (0.26)	0.96 (1.83)	0.034 (1.000)	
	72	0.49 (0.98)	0.25 (0.39)	0.98 (2.47)	0.044 (0.960)	0.21 (0.31)	1.16 (3.10)	0.133 (1.000)	
Resistin^c^	0	1.94 (1.84)	1.00 (0.90)	3.77 (3.78)	0.051 (0.094)	0.85 (0.75)	4.41 (4.49)	0.152 (0.282)	0.244 (0.222)
	24	1.05 (0.98)	0.56 (0.47)	1.98 (2.07)	0.869 (0.966)	0.48 (0.39)	2.29 (2.47)	1.000 (1.000)	
	72	0.81 (0.76)	0.43 (0.35)	1.54 (1.65)	0.502 (0.476)	0.36 (0.29)	1.79 (1.99)	1.000 (1.000)	

Abbreviations: APACHE, Acute Physiology and Chronic Health Evaluation; BMI, body mass index; CI, confidence interval; PAI‐1, plasminogen activator inhibitor‐1; SOFA, Sequential Organ Failure Assessment.

^a^ Lab values rescaled to 10^−6^ power.

^b^ Lab values rescaled to 10^−5^ power.

^c^ Lab values rescaled to 10^−4^ power.

## 4 DISCUSSION

We examined adipokine levels in critically ill patients with acute respiratory failure due to either COVID‐19 or other infectious etiology. Interestingly, adiponectin levels were significantly lower in patients with COVID‐19 respiratory failure than in patients with other forms of infectious respiratory failure, and this remained true even after adjustment for multiple covariates. To our knowledge, this is the first report of adiponectin levels in COVID‐19 ICU patients with acute respiratory failure. van Zelst et al. recently investigated adiponectin levels on emergency room admission in a small subset of patients with and without COVID‐19 (Zelst et al., 2020). They found no difference in admission adiponectin levels between COVID‐19 negative and COVID‐19 positive patients, but adiponectin was only measured at one time point and the cohort mostly consisted of patients who did not require intubation. Caterino et al. recently evaluated adiponectin levels in 53 patients with COVID‐19 infection and found that adiponectin levels were higher in those with severe infection compared to mild or moderate infection, though the difference between the three subgroups was not statistically significant (Caterino et al., 2021).

Adipose tissue is a dynamic endocrine organ with the ability to produce numerous signaling molecules (i.e., adipokines) that impact whole‐body metabolism. Adiponectin is the most abundant adipokine in human plasma, and numerous studies demonstrate its anti‐inflammatory, antioxidative, and insulin‐sensitizing effects (Choi et al., 2020). Obese patients have lower circulating levels of adiponectin (Arita et al., (1999)), and this has been hypothesized to play a role in the poor COVID‐19 outcomes observed in this population (Lockhart & O'Rahilly, 2020). Pre‐clinical studies demonstrate that adiponectin has an anti‐inflammatory function in lung cells (Garcia & Sood, 2012). Furthermore, adiponectin‐deficient mice develop inflammation of the pulmonary vasculature (Summer et al., 2009) and are predisposed to experimental acute lung injury (Konter et al., 2012). These findings suggest that the hypoadiponectinemia frequently seen in obesity could facilitate an exaggerated inflammatory response directed to the pulmonary capillaries (Lockhart & O'Rahilly, 2020).

In the current study, COVID‐19 respiratory failure was associated with significantly reduced adiponectin levels even after adjustment for BMI. These results indicate that COVID‐19 may independently reduce adiponectin in those with respiratory failure, and if true, this holds specific implications for patients with hypoadiponectinemia at baseline (e.g., obesity, type 2 diabetes mellitus, etc.). Alternatively, it could suggest that patients with low adiponectin levels are more prone to develop COVID‐19 respiratory failure.

Some reports suggest potential therapeutic benefit of peroxisome proliferator‐activated receptor‐γ agonists (e.g., pioglitazone) and interleukin‐6 receptor monoclonal antibodies (e.g., tocilizumab) in COVID‐19 patients (Carboni et al., 2020). Interestingly, pioglitazone and tocilizumab each increase circulating levels of adiponectin (Fioravanti et al., 2019; Shimizu et al., 2006). A recent preliminary report from the REMAP‐CAP Investigators found that, in adult ICU patients with COVID‐19 receiving organ support, treatment with the interleukin‐6 receptor monoclonal antibodies tocilizumab and sarilumab improved outcomes (including survival) compared to standard care (Gordon et al., 2021). Other studies with tocilizumab, however, have shown equivocal results (Veiga et al., 2021; Gupta et al., 2021). We now await final results from the numerous clinical trials currently evaluating the efficacy of pioglitazone and tocilizumab as potential COVID‐19 therapies.

Strengths of this study include serial measurement of adipokine levels over 72 hr and comparing COVID‐19 respiratory failure patients to patients with respiratory failure of other infectious etiology. There are several major limitations of this study that should also be noted, including its small sample size. Another limitation is that we restricted our study to only critically ill patients with respiratory failure. Adiponectin levels in COVID‐19 patients with more mild pulmonary disease may differ, so our findings should not be generalized to those whom are not critically ill. Finally, there was no formal matching on demographic/anthropometric variables between cohorts. While the two cohorts were generally similar, the lack of formal matching does warrant caution when interpreting our results.

## 5 CONCLUSION

We conclude that adiponectin levels are reduced in patients with COVID‐19 respiratory failure, even after adjustment for multiple covariates. Larger studies are needed to confirm this report, and we await further work regarding possible therapeutic implications.

## CONFLICT OF INTERESTS

All authors have no conflict of interests to declare or disclose.

## Data Availability

The data that support the findings of this study are available from the corresponding author upon reasonable request.
